# COVID19db: a comprehensive database platform to discover potential drugs and targets of COVID-19 at whole transcriptomic scale

**DOI:** 10.1093/nar/gkab850

**Published:** 2021-09-23

**Authors:** Wenliang Zhang, Yan Zhang, Zhuochao Min, Jing Mo, Zhen Ju, Wen Guan, Binghui Zeng, Yang Liu, Jianliang Chen, Qianshen Zhang, Hanguang Li, Chunxia Zeng, Yanjie Wei, Godfrey Chi-Fung Chan

**Affiliations:** Department of Pediatrics, The University of Hong Kong-Shenzhen Hospital, Shenzhen, Guangdong 518053, China; Shenzhen Institute of Advanced Technology, Chinese Academy of Sciences, Shenzhen, Guangdong 518055, China; Department of Bioinformatics, Outstanding Biotechnology Co., Ltd.-Shenzhen, Shenzhen, China; Shenzhen Institute of Advanced Technology, Chinese Academy of Sciences, Shenzhen, Guangdong 518055, China; Department of Clinical Oncology, The University of Hong Kong-Shenzhen Hospital, Shenzhen, Guangdong 518053, China; School of Information and Software Engineering, University of Electronic Science and Technology of China, Chengdu 610054, China; Department of Bioinformatics, Outstanding Biotechnology Co., Ltd.-Shenzhen, Shenzhen, China; Shenzhen Institute of Advanced Technology, Chinese Academy of Sciences, Shenzhen, Guangdong 518055, China; Center for High Performance Computing, Shenzhen Institutes of Advanced Technology, Chinese Academy of Sciences, Shenzhen, Guangdong 518055, China; CAS Key Laboratory of Health Informatics, Shenzhen Institutes of Advanced Technology, Chinese Academy of Sciences, Shenzhen, Guangdong 518055, China; Department of Bioinformatics, Outstanding Biotechnology Co., Ltd.-Shenzhen, Shenzhen, China; Guangdong Key Laboratory of Animal Conservation and Resource Utilization, Institute of Zoology, Guangdong Academy of Sciences, Guangzhou 510260, China; Department of Bioinformatics, Outstanding Biotechnology Co., Ltd.-Shenzhen, Shenzhen, China; Hospital of Stomatology, Guangdong Provincial Key Laboratory of Stomatology, Guanghua School of Stomatology, Sun Yat-sen University, Guangzhou 510055, China; Shenzhen Institute of Advanced Technology, Chinese Academy of Sciences, Shenzhen, Guangdong 518055, China; Department of Clinical Oncology, The University of Hong Kong-Shenzhen Hospital, Shenzhen, Guangdong 518053, China; Department of Pediatrics, The University of Hong Kong-Shenzhen Hospital, Shenzhen, Guangdong 518053, China; Department of Pediatrics, The University of Hong Kong-Shenzhen Hospital, Shenzhen, Guangdong 518053, China; Department of Pediatrics, The University of Hong Kong-Shenzhen Hospital, Shenzhen, Guangdong 518053, China; Shenzhen Institute of Advanced Technology, Chinese Academy of Sciences, Shenzhen, Guangdong 518055, China; Center for High Performance Computing, Shenzhen Institutes of Advanced Technology, Chinese Academy of Sciences, Shenzhen, Guangdong 518055, China; CAS Key Laboratory of Health Informatics, Shenzhen Institutes of Advanced Technology, Chinese Academy of Sciences, Shenzhen, Guangdong 518055, China; Shenzhen Institute of Advanced Technology, Chinese Academy of Sciences, Shenzhen, Guangdong 518055, China; Center for High Performance Computing, Shenzhen Institutes of Advanced Technology, Chinese Academy of Sciences, Shenzhen, Guangdong 518055, China; CAS Key Laboratory of Health Informatics, Shenzhen Institutes of Advanced Technology, Chinese Academy of Sciences, Shenzhen, Guangdong 518055, China; Department of Pediatrics, The University of Hong Kong-Shenzhen Hospital, Shenzhen, Guangdong 518053, China; Department of Pediatrics and Adolescent Medicine, LKS Faculty of Medicine, The University of Hong Kong, 21 Sassoon Road, Hong Kong 999077, China

## Abstract

Many open access transcriptomic data of coronavirus disease 2019 (COVID-19) were generated, they have great heterogeneity and are difficult to analyze. To utilize these invaluable data for better understanding of COVID-19, additional software should be developed. Especially for researchers without bioinformatic skills, a user-friendly platform is mandatory. We developed the COVID19db platform (http://hpcc.siat.ac.cn/covid19db & http://www.biomedical-web.com/covid19db) that provides 39 930 drug–target–pathway interactions and 95 COVID-19 related datasets, which include transcriptomes of 4127 human samples across 13 body sites associated with the exposure of 33 microbes and 33 drugs/agents. To facilitate data application, each dataset was standardized and annotated with rich clinical information. The platform further provides 14 different analytical applications to analyze various mechanisms underlying COVID-19. Moreover, the 14 applications enable researchers to customize grouping and setting for different analyses and allow them to perform analyses using their own data. Furthermore, a Drug Discovery tool is designed to identify potential drugs and targets at whole transcriptomic scale. For proof of concept, we used COVID19db and identified multiple potential drugs and targets for COVID-19. In summary, COVID19db provides user-friendly web interfaces to freely analyze, download data, and submit new data for further integration, it can accelerate the identification of effective strategies against COVID-19.

## INTRODUCTION

Severe acute respiratory syndrome coronavirus-2 (SARS-CoV-2), SARS-CoV and Middle East respiratory syndrome coronavirus (MERS-CoV) are highly pathogenic betacoronaviruses in the 21st century (1,2). They can result in severe, life-threatening respiratory pathologies and lung injuries (1,2). The ongoing pandemic of SARS-CoV-2, a causative agent of coronavirus disease 2019 (COVID-19), has induced unprecedented impacts globally due to its long incubation period, unpredictably high prevalence due to rapid mutations, and lack of effective intervention strategies (3). Understanding the underlying virus–host interaction at the molecular level and its correlation with the clinical manifestations of COVID-19 is critical for the development of effective prevention and therapy against SARS-CoV-2 and other coronaviruses (1,4).

To facilitate the identification of effective strategies against these coronaviruses, several types of computational resources were developed to provide high resolution coronavirus protein structures (5); validate antibodies and nanoparticles against betacoronavirus (6); identify drug-target interaction of SARS-CoV-2 related infection (7–10); disclose potential immune epitopes of coronaviruses (11); and show global evaluation of SARS-CoV-2 sequences (12). For example, the SARS-CoV-2 3D database aims to understand the coronavirus proteome and evaluate its possible drug targets (7), while the DockCoV2 database was implemented to provide the binding affinity between the approved drugs and seven core proteins related to the SARS-CoV-2 infection (8). Although these useful resources focus on the genetic sequence, protein structure, drug-target interaction, antibody and immune epitopes of SARS-CoV-2 and other coronaviruses, a great effort is still needed to systematically integrate COVID-19 related transcriptomic data and develop comprehensive tools to investigate these data for massive discovering strategies against COVID-19 and its related diseases.

Recently, many COVID-19 related transcriptomic data have been generated and deposited in the National Center for Biotechnology Information Gene Expression Omnibus (NCBI GEO) (13) for public research. However, it is very challenging for the researchers without bioinformatic skills to analyze these invaluable data and their own data to contribute to the drug discovery of COVID-19. For example, these data in the NCBI GEO have great heterogeneity due to their variable origins from different high-throughput platforms, which further hinders their application. In addition, it is also difficult in finding data of specific clinical conditions for study from the massive data in GEO. Thus, there is an urgent need to develop a novel database platform that can systematically integrate the COVID-19 related transcriptomic data, and it can provide comprehensive tools to investigate these integrated data for discovering potential drugs and actionable targets against COVID-19 and its related illnesses.

**Figure 1.**
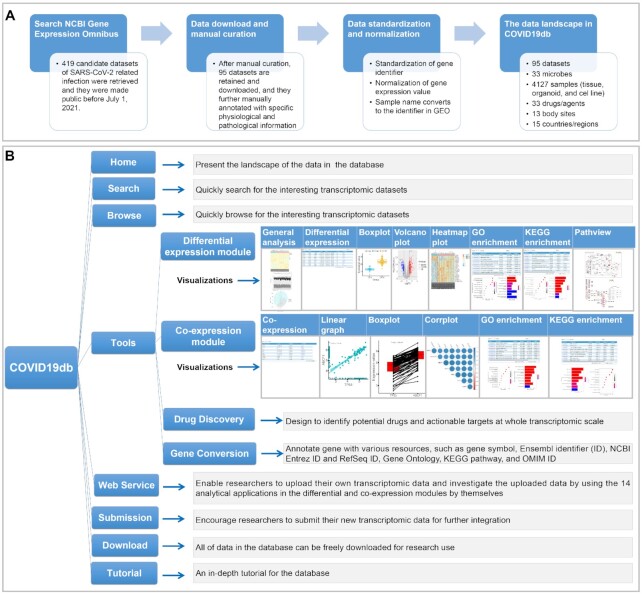
The scheme of data integration and manual curation on the NCBI GEO (**A**) and the web application framework of COVID19db (**B**). GO: Gene Ontology; KEGG: Kyoto Encyclopedia of Genes and Genomes; OMIM: Online Mendelian Inheritance in Man; PCA: Principal Component Analysis.

To overcome these challenges, we implemented the COVID19db (http://hpcc.siat.ac.cn/covid19db & http://www.biomedical-web.com/covid19db/) database platform (Figure 1) which integrated 95 COVID-19 related human transcriptomic datasets in GEO (13), and 39 930 interactions among 2037 drugs, 1116 targets, and 207 pathways in DrugCentral (14) and KEGG (15). These datasets contain transcriptomes of 4127 samples across 13 body sites associated with the exposure of 33 microbes and 33 drugs/agents. Each dataset was manually annotated with rich clinical information and standardized for accurate data retrieval and application development. We further developed 14 different analytical applications (Supplementary Table S1) to analyze these integrated data through customized grouping and setting based on the clinical information of the samples in the dataset. Moreover, COVID19db provides a Web Service tool that allows users to perform the 14 different analyses using their own transcriptomic data. Furthermore, COVID19db provides a Drug Discovery tool to discover potential drugs and actionable targets of COVID-19 and its related diseases at whole transcriptomic scale. As a case study, we used COVID19db and systematically discovered multiple potential drugs and actionable targets of COVID-19 through in-depth investigation on 86 integrated whole blood transcriptomic data. In summary, COVID19db can serve as an important resource to accelerate the discovery and identification of effective strategies against COVID-19 and its related diseases.

## MATERIALS AND METHODS

### Data collection and processing

To collect the COVID-19 related transcriptomic datasets, we searched the NCBI GEO resource (13) for candidate datasets related to the SARS-CoV-2, SARS-CoV and MERS-CoV infection. Search terms and their combinations used in the search strategy included SARS-CoV-2, SARS-CoV, MERS-CoV, COVID-19, SARS and MERS. Totally 419 candidate datasets were retrieved on 1 July 2021. We further manually extracted 95 transcriptomic datasets from the candidate datasets based on three criteria (Figure 1A). Firstly, the dataset contains gene expression profiles of human samples (including tissue, organoid, and cell line) that were exposed with SARS-CoV-2, SARS-CoV, MERS-CoV or their analogues, regardless of which platform the dataset was generated from. In addition, we kept the human transcriptomes related to other microbial exposures in the dataset, which is to facilitate the comparative study between COVID-19 and other infectious diseases. Secondly, the sample and expression data of the dataset can be downloaded for further integration. Thirdly, the single-cell transcriptomic datasets were excluded due to their high complexity compared with the general transcriptomic datasets.

The 95 transcriptomic datasets generated from 15 different high-throughput platforms have great data heterogeneity that hinder them for further applications. To overcome the data heterogeneity, we standardized and normalized the gene identifiers (IDs), sample IDs, and expression values in the datasets by using the R package of org.Hs.eg.db (Supplementary Table S2) and the annotation files in GEO (13). The gene IDs and sample IDs were respectively standardized to the gene symbols in the HUGO Gene Nomenclature Committee (HGNC) (16) and the sample accessions in GEO respectively, while the expression values (such as read counts and fragments per kilobase of exon model per million mapped fragments (FPKM)) in the datasets were standardized to transcripts per million (TPM) and normalized by log2. Approximately 86.32% (82/95), 71.58% (68/95) and 43.16% (41/95) of the datasets were standardized and normalized for the sample IDs, expression values, and gene IDs, respectively. In addition, to enable accurate data retrieval on the database, we manually curated each dataset with rich clinical information, such as microbe, clinical phenotype, drug, age and gender.

### Differential expression module and co-expression module

To comprehensively analyze the integrated data, we implemented 8 differential expression and 6 co-expression analytical applications in the differential expression and co-expression modules, respectively (Figure 1B and Supplementary Table S1). To develop these 14 analytical applications, the R version 4.0.3 environment (https://www.r-project.org/) was established, and the shell and R scripts were used for their development. Details about the R packages used in the 14 applications were described in the Supplementary Table S2 and the ‘Tutorial’ web-page at http://hpcc.siat.ac.cn/covid19db/help.

### Web service

To implement the Web Service tool, we firstly downloaded the annotation file of ‘gencode.v28.annotation.gff3’ in GENCODE (17). Subsequently, we used this annotation file to compute the total exon length of each gene that is further used to standardize and normalize the read counts to TPM. The Web Service tool was implemented in the R project. We described the details of the R packages and resources used in this tool implementation in the Supplementary Tables S2 and S3, respectively. For example, the R packages of org.Hs.eg.db and clusterProfiler are used to convert the Ensembl ID (18), NCBI Entrez ID and RefSeq ID (13) to the gene symbol of HGNC (16). Furthermore, shell scripts were used to encapsulate the Web Service tool, and the tool was deployed in the local server.

### Drug discovery

To implement the Drug Discovery tool, we integrated the drug–target–pathway interactions from the DrugCentral (14) and KEGG PATHWAY (15) resources. We firstly extracted all the drug-target interaction in DrugCentral. And then, we used the R package of org.Hs.eg.db (Supplementary Table S2) to link the drug targeting genes with pathways in KEGG PATHWAY. Based on the integrated interaction data, we further implemented the Drug Discovery tool in the R projects and encapsulated it by shell scripts. Supplementary Tables S2 and S3 illustrate the details of the R packages and resources used in this tool implementation respectively. In addition, we designed links in the resulted table of Drug Discovery analysis to allow users to confirm the drugs, targets, pathways, and their interactions that are associated with COVID-19 and its related diseases through searching keywords of drug, target, pathway, or their combination with ‘SARS-COV OR SARS-COV-2 OR MERS-COV OR COVID-19 OR COVID19 OR MERS OR SARS’ to retrieve the PubMed database (13).

### Gene conversion

The Gene Conversion tool was implemented in the R project, and shell scripts were used to encapsulate this tool. The R packages used for the implementation of the Gene Conversion tool were detailed in the Supplementary Table S2. For example, the R packages of org.Hs.eg.db and clusterProfiler are used to annotate gene with Ensembl ID (18), NCBI Entrez ID and RefSeq ID (13), gene symbol of HGNC (16), GO ID (19), KEGG pathway ID (15) and OMIM ID (20).

### Data storage and web implementation

COVID19db was developed with a separated front-end and back-end framework. The web front-end framework was built with Vue3 (https://vuejs.org), bootStrap4 (https://getbootstrap.com), and JQuery (https://jquery.com), while the web back-end framework was built with the web framework of Spring Boot (https://spring.io/projects/spring-boot). The programs for data processing and application operation were written in Java. The data for the search and browse applications were stored and organized in MySQL. Finally, the database was deployed to the Apache Tomcat web server. The database is publicly accessible through the websites of http://hpcc.siat.ac.cn/covid19db & http://www.biomedical-web.com/covid19db.

## RESULT

### Data content and access

**Figure 2.**
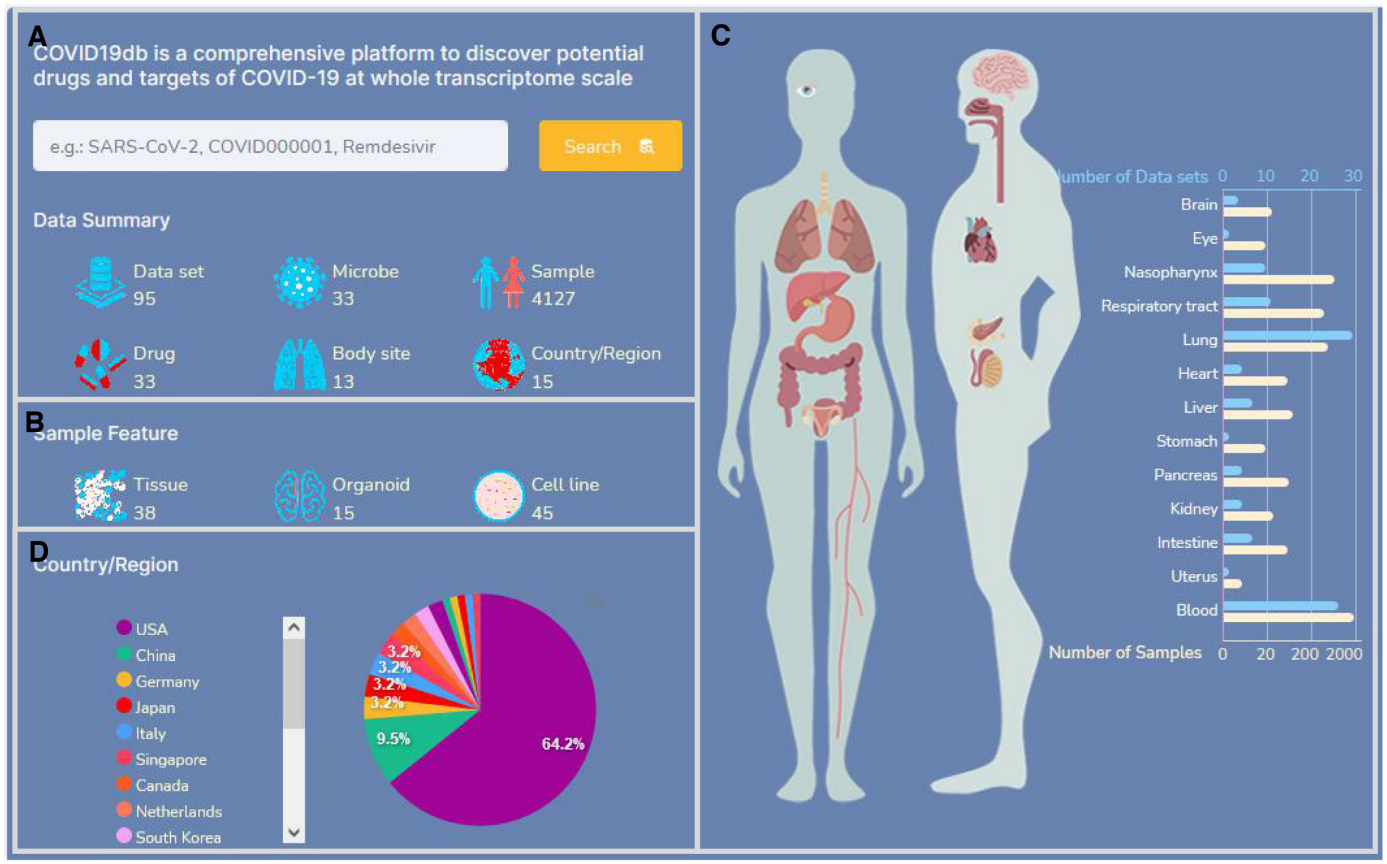
The data landscape and web interface of COVID19db. (**A**) The data summary of the database. (**B**) The number of datasets associated with tissue, organoid, and cell line. (**C**) The number of datasets and samples across 13 body sites. (**D**) The distribution of datasets across countries or regions.

Currently, COVID19db integrated 95 human transcriptomic datasets across 13 body sites and 39 930 drug–target–pathway interactions. These datasets contain 4127 human transcriptomic data related to the exposure of 33 microbes and 33 drugs/agents (Figures 1A and 2A). Of the 95 datasets, 85, 10 and 2 datasets are associated with SARS-CoV-2, MERS-CoV and SARS-CoV, respectively. In addition to these three viruses, the microbes also include influenza viruses, rhinovirus, bacterial, other coronaviruses etc, while the drugs/agents include remdesivir, amlodipine, baricitinib etc. Moreover, 38, 15 and 45 datasets contain the transcriptomic data generated from tissues, organoids, and cell lines, respectively (Figure 2B). In addition, the top six body sites with the largest numbers of datasets are lung, blood, respiratory tract, nasopharynx, liver and intestine (Figure 2C), while the top two countries with the largest contribution to the datasets are USA and China that account for 64.20% (61/95) and 9.50% (9/95), respectively (Figure 2D).

Furthermore, COVID19db is an open access platform that provides user-friendly web interfaces to search, browse, access as well as download data and submit new data for further integration (Figure 1B). For example, COVID19db provides the search application that enables users to retrieve the interesting data with various filtering parameters such as microbe, drug, sample name and type, and clinical phenotype (Supplementary Figure S1). The search application also offers a smart assistance function to enter a keyword to list the entries closest to that expectation. At the ‘Home’ web-page of the database, organs in the human diagram can be clicked to browse their entry details (Figure 2C). All of the search and browse results are presented in a user-friendly table (Supplementary Figure S1). Moreover, the COVID19db IDs in the table (Supplementary Figure S1) are able to navigate to further web-pages for more details about the entry and access to the differential expression and co-expression modules (e.g.: http://hpcc.siat.ac.cn/covid19db/searchDetail?id=COVID000010).

In addition, all data in COVID19db are available as open access and can be downloaded for research freely. The database provides a variety of common data formats for download, including TABLE, CSV and JSON (http://hpcc.siat.ac.cn/covid19db/download). Moreover, users are encouraged to submit their new transcriptomic data associated with SARS-CoV-2, SARS-CoV, MERS-CoV and other related viruses. Once the data quality and ethical approval status were checked and approved by our submission committee, the submission data will be adopted in the future version (http://hpcc.siat.ac.cn/covid19db/submit).

### Comprehensive analysis on the integrated and researcher's own transcriptomic data

Researchers without bioinformatic skills will have difficulty to analyze the integrated transcriptomic data in COVID19db. Therefore, COVID19db provides a user-friendly analytical platform for researchers to retrieve and analyze the integrated data for mechanistic study and drug screening on the SARS-CoV-2 related infection. Importantly, all 14 analytical applications mentioned allow custom grouping and setting to perform the analysis individually based on the clinical information of the samples (Supplementary Figure S2). Details of the 14 analytical applications are described in Figure 1B, Supplementary Table S1, and the ‘Tutorial’ web-pages on the database.

To better serve the research community, the Web Service tool is designed to enable researchers in conducting in-depth investigation on their own human transcriptomic data. This tool is a systematic pipeline that can integrate the uploaded transcriptomic data automatically. Then it will assign a temporary identifier to the uploaded data for further analysis. After the data integration completed, a new web-page will pop up to allow investigators to conduct the 14 analyses on their uploaded data privately. Considering that different types of gene ID and expression value were used in the uploaded data of different researchers due to the data generated by different platforms, the Web Service tool is designed to handle various types of gene ID (including gene symbol in HGNC (16), Ensembl ID (18), NCBI Entrez ID and RefSeq ID (13)) and expression value (such as read counts, TPM, and FPKM). To better protect the data security, the Web Service tool of COVID19db allows users to delete their uploaded data at any stage using the assigned temporary data identifier, and it regularly cleans up the uploaded data monthly.

Although the whole transcriptomic analysis can identify hundreds or even thousands of significant differential genes between cases and their controls, it is very challenging to identify potential drugs and actionable targets based on such large-scale genetic information. To cope with this challenge, we developed the Drug Discovery tool in COVID19db to discover and prioritize the potential drugs and actionable targets of COVID-19 at whole transcriptomic scale based on the integrated data and the differential expression application in the differential expression module. Moreover, this tool not only enables researchers to input thousands of genes identified by themselves, but also allows them to select the types of the inputted gene ID, such as the gene symbol, Ensembl ID, NCBI Entrez ID, and RefSeq ID. As all drug-target interactions in DrugCentral and KEGG PATHWAY were integrated, the Drug Discovery tool can be used to discover and identify potential drugs and targets of all kinds of human diseases, not just COVID-19, at whole transcriptome scale. Taking into account the different gene nomenclatures used in different resources, COVID19db provides the Gene Conversion tool to convert different gene IDs among different resources and annotate them with GO (19), KEGG pathway (15), and OMIM (20) IDs.

Finally, the results from the above applications and tools are visualized as graph and/or table. The graphic results can be freely downloaded and saved as a PDF or PNG file with high resolution. By right clicking on the table body, an instruction form will be displayed to copy and export the results for further analysis (Supplementary Figure S3). Moreover, each table can be sorted by columns, and it provides a filter box for quick search. In addition, the menu at the table header is designed to fit columns and rows to display the interesting data (Supplementary Figure S3). For more information, a detail tutorial is available for the database at website of http://hpcc.siat.ac.cn/covid19db/help.

### Case study: using COVID19db to discover potential drugs and actionable targets of COVID-19 at whole transcriptomic scale

**Figure 3.**
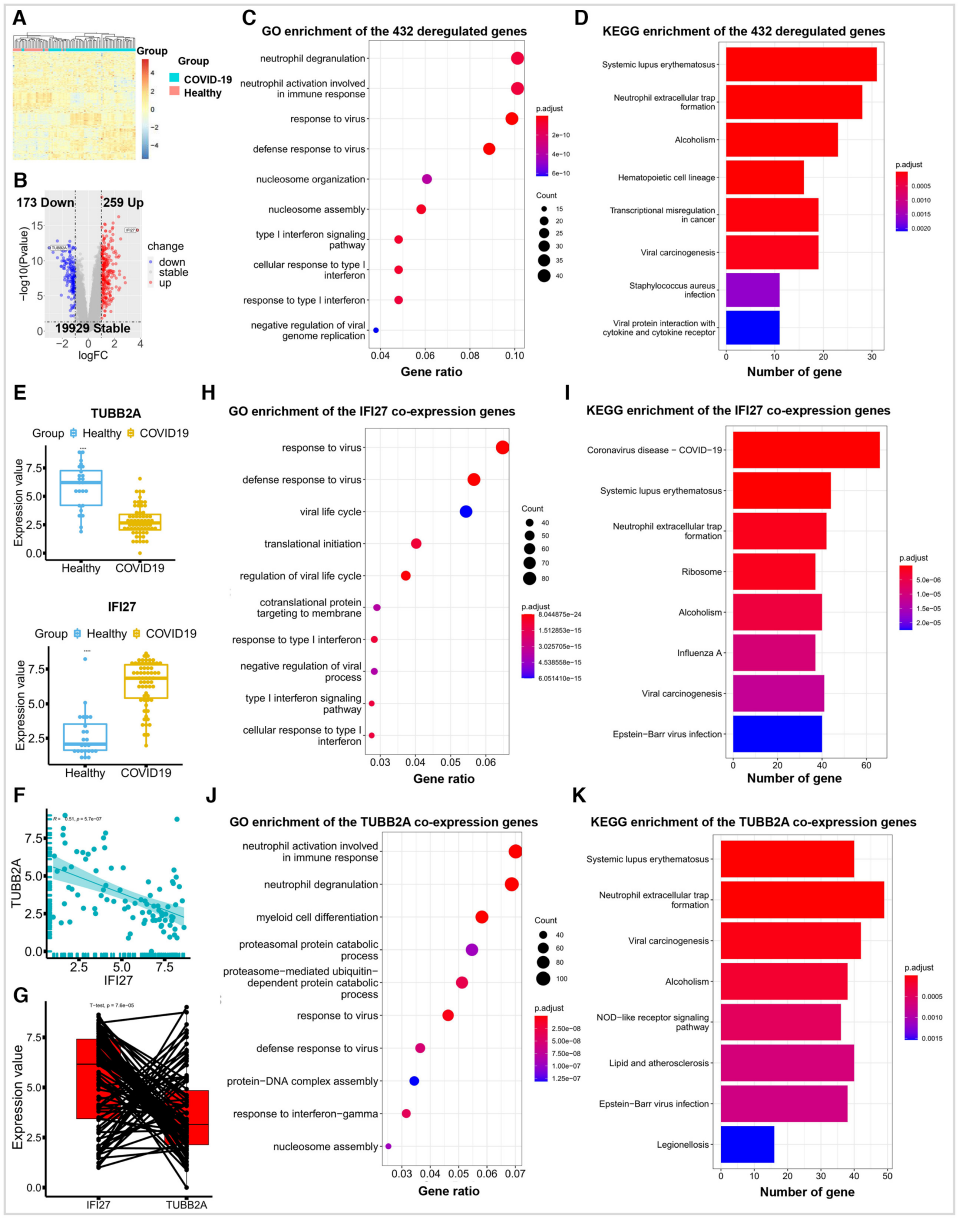
Comprehensively investigation on the whole blood transcriptomic data of 24 healthy controls and 62 COVID-19 patients. (**A**) The heat map showed different gene expression patterns between the two groups. (**B**) 432 dysregulated (|logFC| ≥ 1 & *P*-value ≤ 0.05) genes were identified, including 259 up- and 173 down-regulated genes through the differential expression and volcano plot analysis in the differential expression module. The volcano plot also indicated that IFI27 and TUBB2A are the top ranked genes in the up- and down-regulated genes, respectively. (**C**, **D**) The GO and KEGG enrichment results of the dysregulated gene. (**E**) The plots indicated that the IFI27 and TUBB2A genes were dysregulated after the SARS-CoV-2 infection. ****P-value ≤ 0.0001. (**F**, **G**) TUBB2A and IFI27 showed significantly negative correlation both in paired and unpaired methods. (**H**–**K**). The GO and KEGG enrichment results of IFI27 and TUBB2A based on them co-expression genes (|correlation cutoff| ≥ 0.4 & correlation *P*-value cutoff ≤ 0.01).

To discover the potential drugs and actionable targets of COVID-19, we systematically investigated the whole blood transcriptomic data of 24 healthy controls and 62 COVID-19 patients (COVID19db ID: COVID000010) (21). Firstly, the results showed that COVID-19 patients have significantly different gene expression patterns to their controls (Figure 3A & Supplementary Figure S4). We further identified 432 dysregulated genes between the two groups (|logFC| ≥ 1 & *P*-value ≤ 0.05), including 259 up- and 173 down-regulated genes (Figure 3B). Moreover, the GO and KEGG enrichment results indicated that these dysregulated genes were enriched on several vital signaling pathways associated with antiviral immunity (22–24) (Figure 3C, D). In addition, it is found that the top ranked genes in the up- and down-regulated genes are IFI27 and TUBB2A respectively (Figure 3B and E), and they showed significantly negative correlation (Figure 3F, G). The up-regulation of IFI27 associates with COVID-19 symptomatic infection, which was confirmed by recent studies (25,26), but whether the down-regulation of TUBB2A associates with similar clinical scenario requires further confirmation.

**Figure 4.**
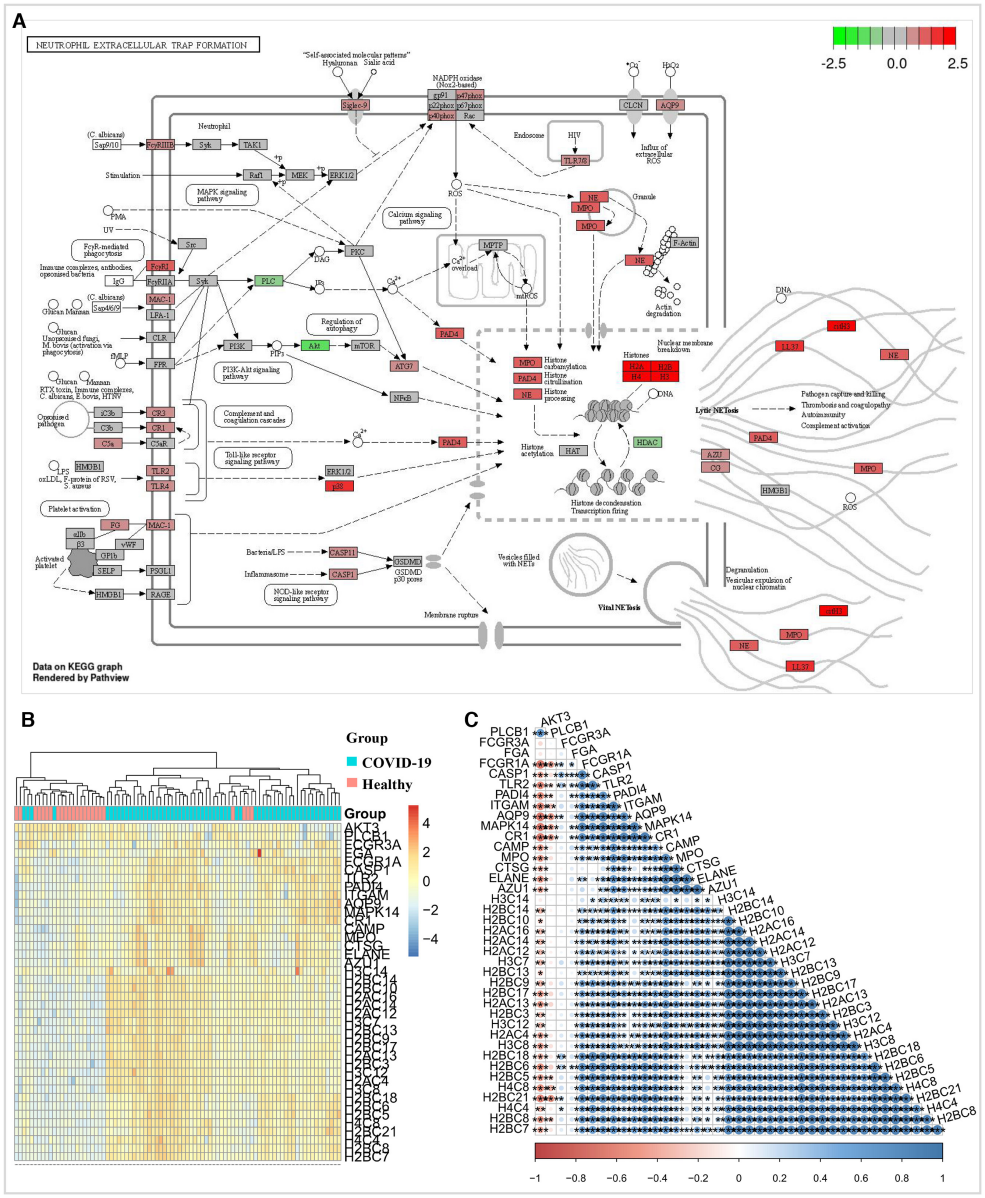
Neutrophil extracellular trap formation pathway was activated after the SARS-CoV-2 infection. (**A**) The pathview result showed that most of genes associated with neutrophil extracellular trap (NET) formation were up-regulated in the whole blood cells of COVID-19 patients compared with those in their controls. The color bar indicates the range of log FC value. (**B**) The heat map suggested that the NET formation associated genes can significantly distinguished the COVID-19 patients from their controls. (**C**) The corrplot result indicated that most of the NET formation associated genes have significantly correlations with each other. *: *P*-value ≤ 0.05; **: *P*-value ≤ 0.01; ***: *P*-value ≤ 0.001.

Furthermore, the function enrichment results of the IFI27 and TUBB2A co-expression genes are both consistent with those enrichment results of the dysregulated genes (Figure 3C, D and H–K), suggesting that the two genes may play a critical role in regulating antiviral immunity pathways of the SARS-Cov-2 infection, such as the KEGG pathway responsible for neutrophil extracellular trap (NET) formation that was widely accepted to be contributory to the COVID-19 pathology (23,27–29). Consistent with these published results, our pathway analysis further confirmed that NET formation in whole blood cells were obviously activated after the SARS-CoV-2 infection (Figure 4A and Supplementary Figure S5A). Moreover, the heat map results also suggested that the genes associated with NET formation can clearly distinguish the COVID-19 patients from their controls (Figure 4B), and most of these genes showed significant correlations with each other (Figure 4C). It is also noted that the 432 dysregulated genes, the IFI27 and TUBB2A co-expression genes were consistently enriched in the disease pathway of systemic lupus erythematosus (Figure 3D, I and K), which was also activated in the COVID-19 patients compared with their controls (Supplementary Figure S5B). Therefore, we concluded that the IFI27, TUBB2A and the pathways of NET formation and lupus like immune profile may be the potential immune deregulation mechanism triggered by COVID-19, but further experimental validation *in vitro* and *in vivo* is needed.

**Figure 5.**
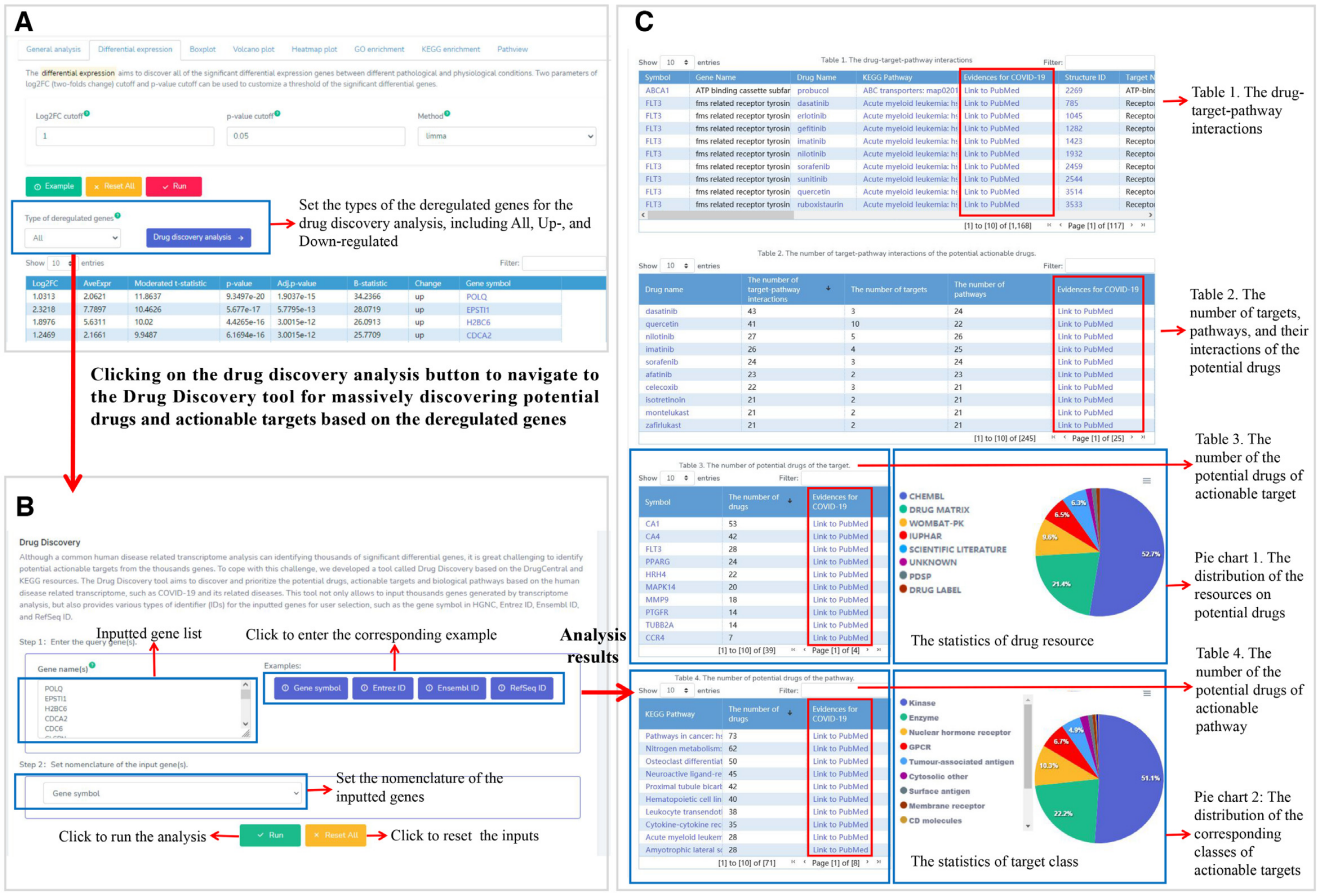
The application workflow and web interface of the Drug Discovery tool. (**A**) 432 dysregulated genes were identified using the differential expression application. Further clicking on the drug discovery analysis button can navigate to the Drug Discovery tool for the potential drugs of COVID-19 to target these dysregulated genes. (**B**) The web interface of the Drug Discovery tool. (**C**) The tables and pie charts resulted from the Drug Discovery tool showed the landscape of the prioritized potential drugs, actionable targets and biological pathways of COVID-19 based on the 432 dysregulated genes. Users can click on the ‘Link to PubMed’ links to further retrieve PubMed for publication evidences to confirm the drugs, targets, pathways, and their interactions that are associated with COVID-19 and its related diseases.

To discover potential drugs and more actionable targets against COVID-19, we further used the Drug Discovery tool to investigate the 432 dysregulated genes and identified 39 of which are associated with 245 drugs and 71 pathways to form 1168 interactions (Figure 5A, B and Table 1 in Figure 5C). The top three drugs with the largest number of interactions are dasatinib, quercetin and nilotinib (Table 2 in Figure 5C), the top three actionable targets with the largest number of potential drugs are CA1, CA4 and FLT3 (Table 3 in Figure 5C), and the top three actionable pathway terms are pathways in cancer, nitrogen metabolism and osteoclast differentiation (Table 4 in Figure 5C). In addition, the two pie charts in the Figure 5C presented the distributions of the resources on potential drugs and their corresponding classes of actionable targets. We suggest users click on links called ‘Link to PubMed’ in Figure 5C to further retrieve PubMed (13) for publication evidences to confirm the drugs, targets, pathways and their interactions that are associated with COVID-19 and its related diseases. By further investigation the 1168 drug–target–pathway interactions (Table 1 in Figure 5C), we identified four drugs (including bortezomib, boceprevir, sivelestat and telaprevir) from the 245 potential drugs and these four drugs can target the human leukocyte elastase (ELANE) to inhibit lupus related pathway (Supplementary Table S4), which was associated with COVID-19 (Figure 3D, I and K) and was activated in patients with the SARS-CoV-2 infection (Supplementary Figure S5B). The anti-viral activity of these four drugs against COVID-19 has been proposed by several latest studies (30–36). For example, both two recent studies suggested that boceprevir can effectively inhibits SARS-CoV-2 by targeting its main protease (31,32). In summary, this case study demonstrated that COVID19db can serve as an important resource to facilitate drug discovery of effective strategies against COVID-19 and its related disease.

## DISCUSSION

As far as we know, COVID19db is the first database platform that is specifically designed to discover the potential drugs and actionable targets of COVID-19 at whole transcriptomic scale. Both in terms of data content and platform functions, COVID19db is significantly different from the recently published SARS-CoV-2 related databases (such as GESS (12), SARS-CoV-2 3D database (7), CoV3D (5), DockCoV2 (8), CoV-AbDab (6) and COVIEdb (11)), which focus on providing genetic sequence, protein structure, drug-target interaction, antibody, and immune epitopes of SARS-CoV-2 and other coronaviruses. For example, DockCoV2 focuses on predicting the binding affinity of approved drugs for seven proteins (8), while COVID19db aims to discover and identify potential drugs and actionable targets of COVID-19 and its related diseases at whole transcriptomic scale.

In the unpublished COVID-19 related resources, it is found that only the CovidExpress (37) database integrates the COVID-19 related transcriptomic data from GEO, which is posted on the *bioRxiv* preprint server on 26 May 2021 (37). Compared with CovidExpress, COVID19db is significantly different and/or superior in terms of data content and utilities (Supplementary Table S5). Firstly, 82.53% (3406/4127) and 76.84% (73/95) of the transcriptomes and datasets were uniquely collected in COVID19db respectively (Supplementary Table S5). In addition, COVID19db provides 39,930 drug–target–pathway interactions, which are missing in CovidExpress (Supplementary Table S5). Secondly, COVID19db provides six co-expression analytical functions to investigate the integrated transcriptomic data, while CovidExpress only supports one of those functions (Supplementary Table S5). Thirdly, COVID19db provides five additional tools, but four of which are not included in the CovidExpress database (Supplementary Table S5). For example, we used the Drug Discovery tool and identified multiple potential drugs and actionable targets of COVID-19 at whole transcriptomic scale based on the integrated data in COVID19db (Figure 5 and Supplementary Table S4). Moreover, the Web Service tool allows researchers to conduct all analyses in the database using their own human transcriptomic data (Supplementary Table S5). Finally, most analyses and result visualizations in CovidExpress rely on the Python 3.6+ environment established by user themselves to install the cellxgene tool, which is not needed by COVID19db. COVID19db is a user-friendly web database, and it works well in different browsers, such as Google Chrome, Firefox, Microsoft Edge and Apple Safari (Supplementary Table S5). Taken together, COVID19db database is significantly different to existing databases and is superior to other related databases, both published and unpublished.

More and more COVID-19 related transcriptomic datasets are expected to be published. Therefore, COVID19db will continue to integrate the newly published data in the public resources such as NCBI GEO (13) by expanding the database every 6 months. As different datasets integrated in the database were generated by different studies, high throughput platforms, and analysis pipelines, we currently suggest researchers to perform gene expression analyses within individual dataset. To cope with this challenge, we will improve the analyses to adjust for batch effects between different datasets in the future. Moreover, our initial efforts were focused on the human transcriptomic data related to the SARS-CoV-2, SARS-CoV and MERS-CoV infection due to the vast amount of the existing transcriptomic data and the best genome annotation. Consequently, we also plan to expand the database to include more coronaviruses and their host organisms with the available multiple-omics data (such as single-cell transcriptomics, genomics, epigenetics, metagenomics and proteomics data) in the future releases. At the same time, we will develop new web functions to analyze and visualize these new omics data, and we will also integrate more computational tools and methods (10,38–42) for assisting the identification of effective strategies against COVID-19 and its related diseases. Furthermore, we plan to integrate and curate experimentally validated association data between drugs and human targets related to SARS-CoV-2 infection from publications and further add evidence to verify whether certain drugs are really against its infection. We believe that these additional data and functionalities will significantly increase the application efficiency of COVID19db in the COVID-19 research community.

## DATA AVAILABILITY

The COVID19db platform is accessible at websites of http://hpcc.siat.ac.cn/covid19db and http://www.biomedical-web.com/covid19db.

## Supplementary Material

gkab850_Supplemental_File
