# Correction to ‘COVID19db: a comprehensive database platform to discover potential drugs and targets of COVID-19 at whole transcriptomic scale’

**DOI:** 10.1093/nar/gkad646

**Published:** 2023-07-28

*Nucleic Acids Research*, Volume 50, Issue D1, 7 January 2022, Pages D747–D757, https://doi.org/10.1093/nar/gkab850

In the originally published version of this manuscript, there is an error in the Funding section.

Grant number ‘RCYX2020071411473419’ should have been given as ‘RCYX20200714114734194’.

These details have been corrected only in this correction notice to preserve the published version of record.

