# Supplementary material for: COVID19db: a comprehensive database platform to discover potential drugs and targets of COVID-19 at whole transcriptomic scale
Source: Nucleic Acids Res. 2021 Sep 23;50(D1):D747–57. doi: 10.1093/nar/gkab850 (PMC8728200; doi:10.1093/nar/gkab850)
Supplement: gkab850_Supplemental_File [file gkab850_supplemental_file.pdf]

Supplementary data of COVID19db

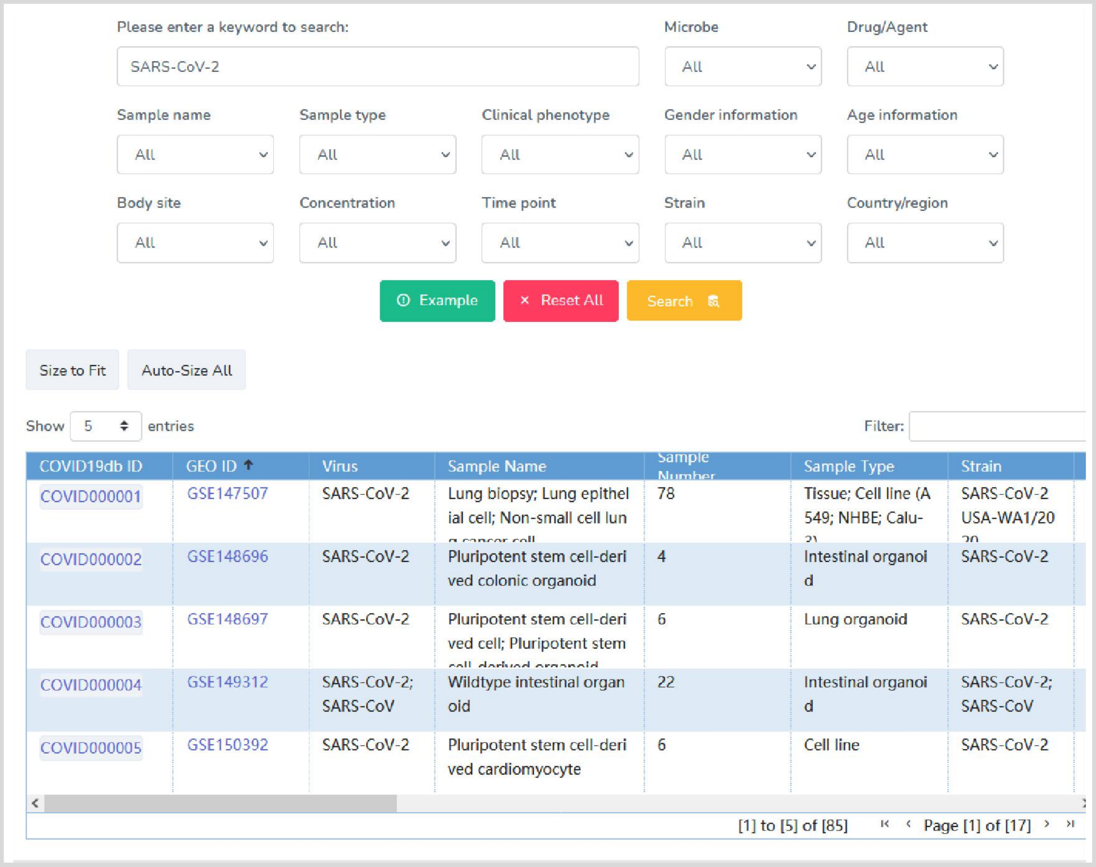

Supplementary Figure S1. The web interface of the search application on COVID19db.

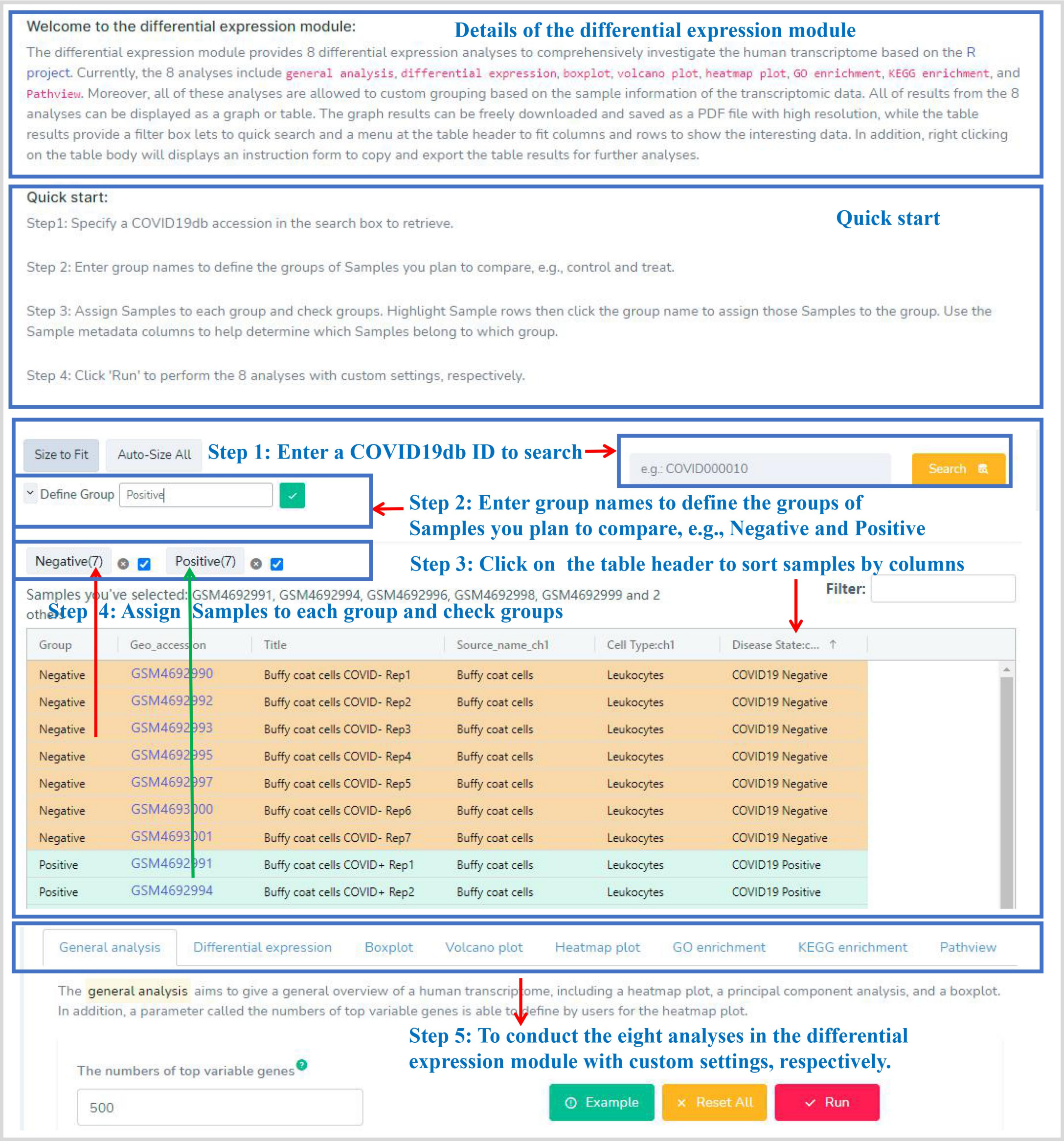

Supplementary Figure S2. An example of customized grouping and setting for the analytical applications in the differential expression and co-expression modules.

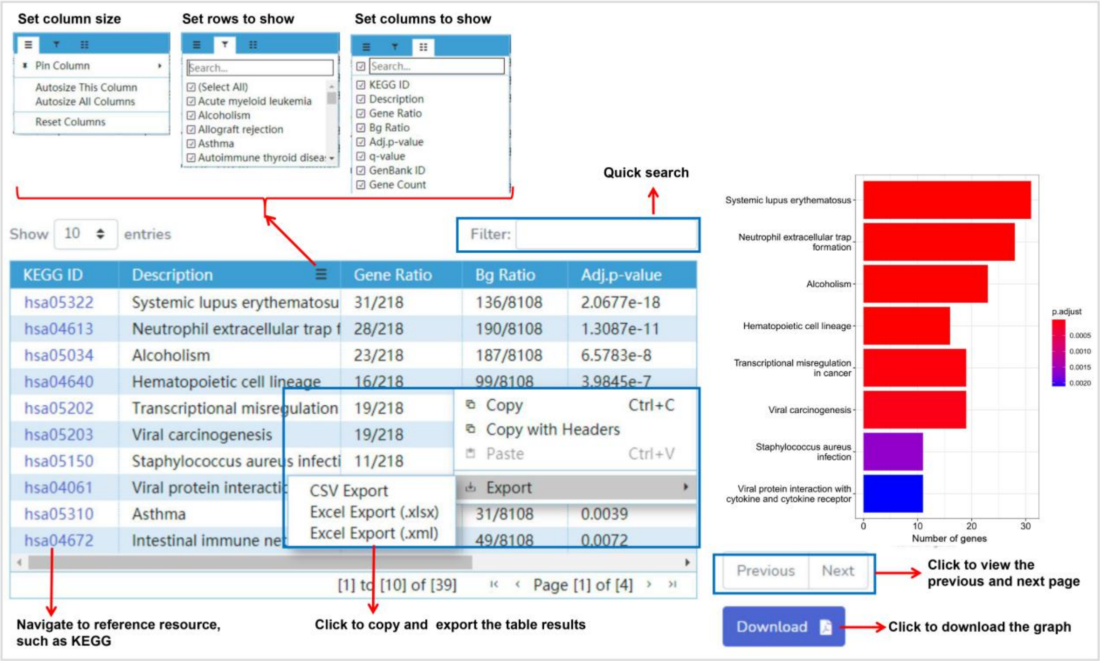

Supplementary Figure S3. The web interface of the resulted table and graph in the COVID19db database platform.

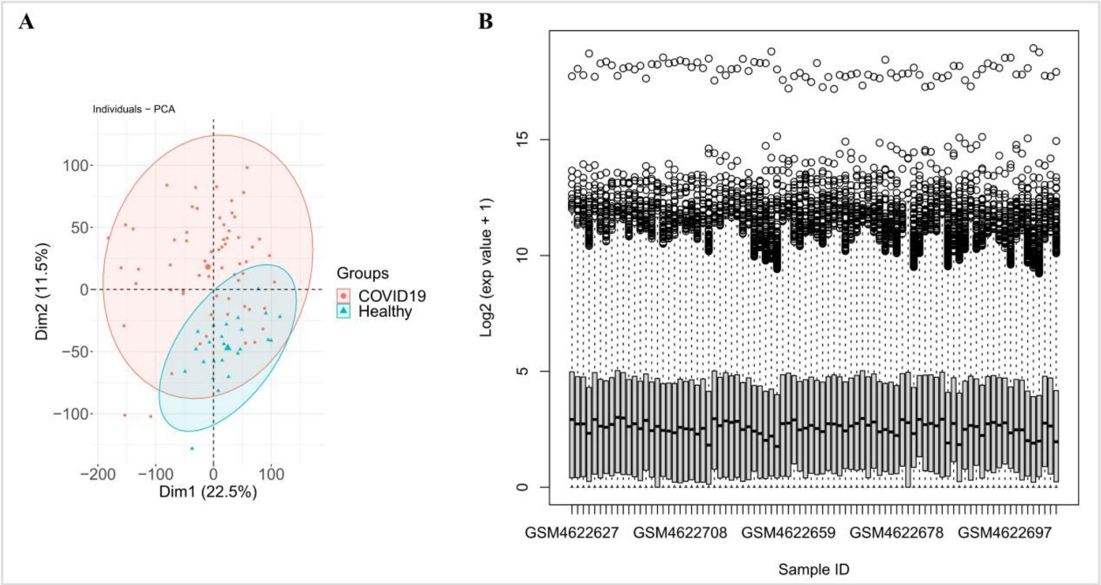

Supplementary Figure S4. The results of the general analysis on the COVID000010 dataset in COVID19db, including the principal component analysis (A) and boxplot (B).

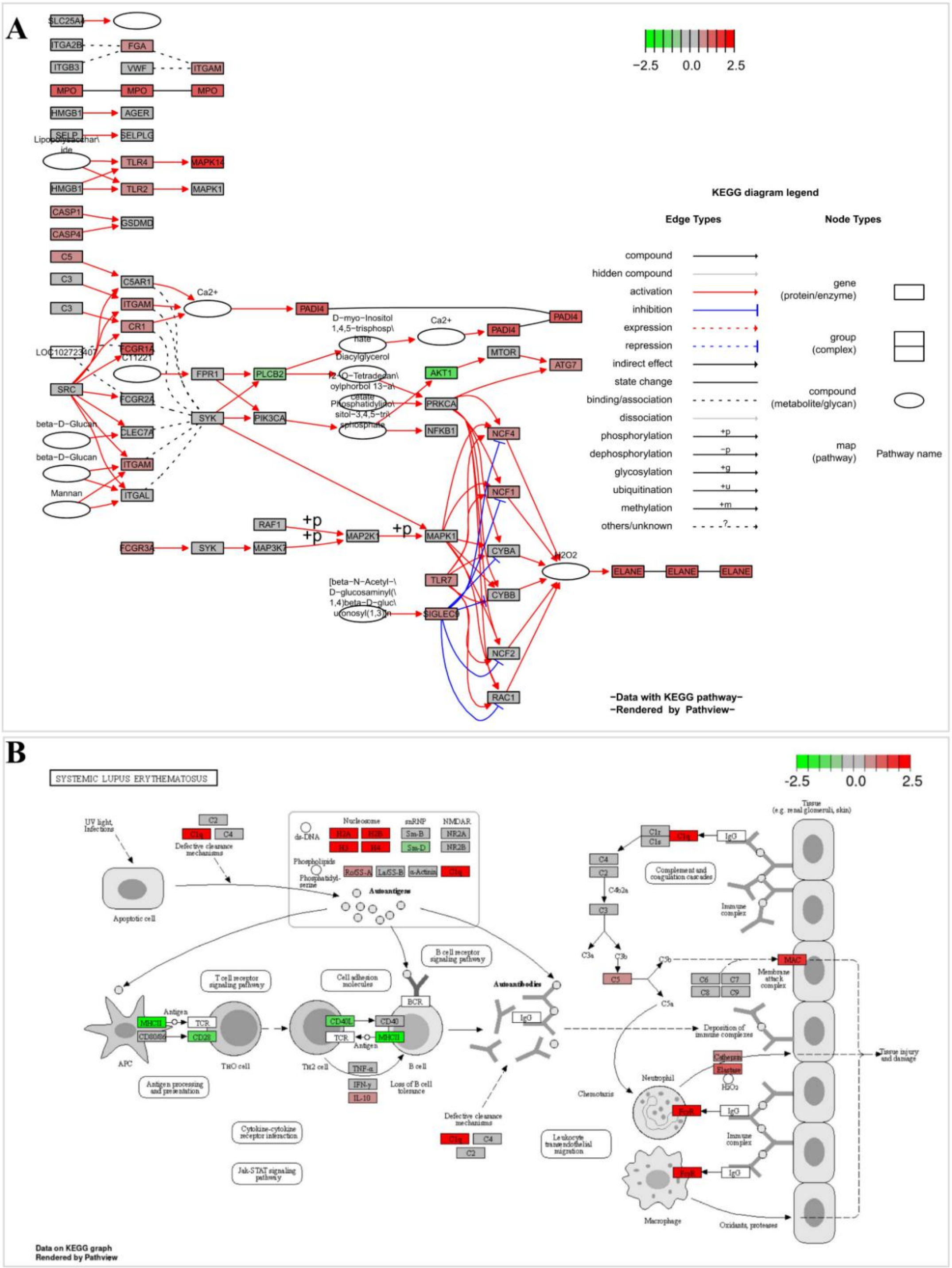

Supplementary Figure S5. Neutrophil extracellular trap formation and systemic lupus erythematosus related pathways were activated in the whole blood cells of COVID-19 patients compared with those in their controls. (A) The pathview result of neutrophil extracellular trap formation with the KEGG native parameter set with ‘No’. (B) The pathview result of systemic lupus erythematosus with the KEGG native parameter set with ‘Yes’. The color bar indicates the range of logFC value of genes.

Supplementary Table S1. The description of the 14 analytical applications in the differential expression and co-expression modules

Analysis application	Description
**Differential expression module**	
General analysis	To give a general overview of a transcriptomic dataset, including heat map plot, principal component analysis, and box plot.
Differential expression	To identify differential genes between two conditions.
Boxplot	To draw a box plot to present a gene expression difference in different conditions.
Volcano plot	To draw a volcano diagram to depict the gene difference between two conditions.
Heatmap plot	To display gene expression pattern in different conditions.
GO enrichment	To conduct the gene ontology enrichment analysis on the significant differential genes between two conditions.
KEGG enrichment	To conduct the KEGG pathway enrichment analysis on the significant differential genes.
Pathview	To present the gene expression patterns in a specific KEGG signaling pathway.
**Co-expression module**	
Co-expression	To compute the correlations of an interesting gene with all genes in a specific condition.
Linear graph	To perform correlation analysis in a specific condition and draw a linear graph.
Boxplot	To conduct a paired correlation analysis for two genes in a specific condition and draw a box plot.
Corrplot	To compute the correlationships among multiple genes in a specific condition and draw a corrplot.
GO enrichment	To identify the function of an interesting gene in a specific condition based on the annotations of its co-expression genes in the GO resource.
KEGG enrichment	To identify the function of an interesting gene in a specific condition based on the annotations of its co-expression genes in the KEGG resource.

Supplementary Table S2. The details of the R packages used in COVID19db

Web application	Package name	Description	Version	Reference	URL
**Differential expression module**					
General analysis	dplyr	Working with data frame like objects.	v 1.0.5	Hadley Wickham *et al.* (2021)	https://cran.r-project.org/web/packages/dplyr
	FactoMineR	Dedicated to multivariate exploratory data analysis.	v 1.08	Sebastien Le *et al.* (2008)	http://factominer.free.fr/citation.html
	factoextra	Easy multivariate data analyses and elegant visualization.	v 1.07	Alboukadel Kassambara and Fabian Mundt (2020)	https://cran.r-project.org/web/packages/factoextra
	ggplot2	Elegant graphics for data analysis.	v 2016	H. Wickham (2016)	https://ggplot2.tidyverse.org
	pheatmap	Plotting pretty heatmaps.	v 1.0.12	Raivo Kolde (2019)	https://cran.r-project.org/web/packages/pheatmap
Differential expression	dplyr	Working with data frame like objects.	v 1.0.5	Hadley Wickham *et al.* (2021)	https://cran.r-project.org/web/packages/dplyr
	jsonlite	A practical and consistent mapping between JSON data and R objects.	v 2014	Jeroen Ooms (2014)	https://arxiv.org/abs/1403.2805
	limma	Powers differential expression analyses for RNA-sequencing and microarray studies.	v 2015	Matthew E Ritchie *et al.* (2015); PMID: 25605792	http://www.bioconductor.org/packages/release/bioc/html/limma.html
Boxplot	dplyr	Working with data frame like objects.	v 1.0.5	Hadley Wickham *et al.* (2021)	https://cran.r-project.org/web/packages/dplyr
	ggpubr	“ggplot2” based publication ready plots.	v 0.4.0	Alboukadel Kassambara (2020)	https://cran.r-project.org/web/packages/ggpubr
	reshape2	Reshaping data.	v 1.4.4	Hadley Wickham (2007)	https://cran.r-project.org/web/packages/reshape2
Volcano plot	dplyr	Working with data frame like objects.	v 1.0.5	Hadley Wickham *et al.* (2021)	https://cran.r-project.org/web/packages/dplyr
	ggplot2	Elegant graphics for data analysis.	v 2016	H. Wickham (2016)	https://ggplot2.tidyverse.org
	limma	Powers differential expression analyses for RNA-sequencing and microarray studies.	v 2015	Matthew E Ritchie *et al.* (2015); PMID: 25605792	http://www.bioconductor.org/packages/release/bioc/html/limma.html
Heatmap plot	dplyr	Working with data frame like objects.	v 1.0.5	Hadley Wickham *et al.* (2021)	https://cran.r-project.org/web/packages/dplyr
	ggplot2	Elegant graphics for data analysis.	v 2016	H. Wickham (2016)	https://ggplot2.tidyverse.org
	jsonlite	A practical and consistent mapping between JSON data and R objects.	v 2014	Jeroen Ooms (2014)	https://arxiv.org/abs/1403.2805
	pheatmap	Plotting pretty heatmaps.	v 1.0.12	Raivo Kolde (2019)	https://cran.r-project.org/web/packages/pheatmap
GO enrichment	clusterProfiler	Statistical analysis and visualization of functional profiles	v 3.18.1	Guangchuang Yu *et al.* (2012); PMID:	http://www.bioconductor.org/packages/release/bioc/html/clus

		for genes and gene clusters.		22455463	terProfiler.html
	dplyr	Working with data frame like objects.	v 1.0.5	Hadley Wickham *et al.* (2021)	https://cran.r-project.org/web/packages/dplyr
	ggplot2	Elegant graphics for data analysis.	v 2016	H. Wickham (2016)	https://ggplot2.tidyverse.org
	jsonlite	A practical and consistent mapping between JSON data and R objects.	v 2014	Jeroen Ooms (2014)	https://arxiv.org/abs/1403.2805
	limma	Powers differential expression analyses for RNA-sequencing and microarray studies.	v 2015	Matthew E Ritchie *et al.* (2015); PMID: 25605792	http://www.bioconductor.org/packages/release/bioc/html/limma.html
	org.Hs.eg.db	Genome wide annotation for Human.	v 3.11.4	Marc Carlson (2020)	http://www.bioconductor.org/packages/release/data/annotation/html/org.Hs.eg.db.html
	Rgraphviz	Graph for R objects.	v 2.32.0	Kasper Daniel Hansen *et al.* (2020)	http://www.bioconductor.org/packages/release/bioc/html/Rgraphviz.html
	stringr	Common string operations.	v 1.4.0	Hadley Wickham (2019)	https://cran.r-project.org/web/packages/stringr
	topGO	Enrichment analysis for Gene Ontology.	v 2.40.0	Adrian Alexa and Jorg Rahnenfuhrer (2020)	http://www.bioconductor.org/packages/release/bioc/html/topGO.html
KEGG enrichment	clusterProfiler	Statistical analysis and visualization of functional profiles for genes and gene clusters.	v 3.18.1	Guangchuang Yu *et al.* (2012); PMID: 22455463	http://www.bioconductor.org/packages/release/bioc/html/clusterProfiler.html
	dplyr	Working with data frame like objects.	v 1.0.5	Hadley Wickham *et al.* (2021)	https://cran.r-project.org/web/packages/dplyr
	ggplot2	Elegant graphics for data analysis.	v 2016	H. Wickham (2016)	https://ggplot2.tidyverse.org
	jsonlite	A practical and consistent mapping between JSON data and R objects.	v 2014	Jeroen Ooms (2014)	https://arxiv.org/abs/1403.2805
	limma	Powers differential expression analyses for RNA-sequencing and microarray studies.	v 2015	Matthew E Ritchie *et al.* (2015); PMID: 25605792	http://www.bioconductor.org/packages/release/bioc/html/limma.html
	org.Hs.eg.db	Genome wide annotation for Human.	v 3.11.4	Marc Carlson (2020)	http://www.bioconductor.org/packages/release/data/annotation/html/org.Hs.eg.db.html
	pathview	Pathway-based data integration and visualization.	v 1.30.1	Luo Weijun and Brouwer Cory (2014); PMID: 23740750	http://www.bioconductor.org/packages/release/bioc/html/pathview.html
	Rgraphviz	Graph for R objects.	v 2.32.0	Kasper Daniel Hansen *et al.* (2020)	http://www.bioconductor.org/packages/release/bioc/html/Rgraphviz.html
	stringr	Common string operations.	v 1.4.0	Hadley Wickham (2019)	https://cran.r-project.org/web/packages/stringr

Pathview	clusterProfiler	Statistical analysis and visualization of functional profiles for genes and gene clusters.	v 3.18.1	Guangchuang Yu *et al.* (2012); PMID: 22455463	http://www.bioconductor.org/packages/release/bioc/html/clusterProfiler.html
	dplyr	Working with data frame like objects.	v 1.0.5	Hadley Wickham *et al.* (2021)	https://cran.r-project.org/web/packages/dplyr
	ggplot2	Elegant graphics for data analysis.	v 2016	H. Wickham (2016)	https://ggplot2.tidyverse.org
	jsonlite	A practical and consistent mapping between JSON data and R objects.	v 2014	Jeroen Ooms (2014)	https://arxiv.org/abs/1403.2805
	limma	Powers differential expression analyses for RNA-sequencing and microarray studies.	v 2015	Matthew E Ritchie *et al.* (2015); PMID: 25605792	http://www.bioconductor.org/packages/release/bioc/html/limma.html
	org.Hs.eg.db	Genome wide annotation for Human.	v 3.11.4	Marc Carlson (2020)	http://www.bioconductor.org/packages/release/data/annotation/html/org.Hs.eg.db.html
	pathview	Pathway-based data integration and visualization.	v 1.30.1	Luo Weijun and Brouwer Cory (2014); PMID: 23740750	http://www.bioconductor.org/packages/release/bioc/html/pathview.html
	stringr	Common string operations.	v 1.4.0	Hadley Wickham (2019)	https://cran.r-project.org/web/packages/stringr
**Co-expression module**					
Co-expression	dplyr	Working with data frame like objects.	v 1.0.5	Hadley Wickham *et al.* (2021)	https://cran.r-project.org/web/packages/dplyr
	jsonlite	A practical and consistent mapping between JSON data and R objects.	v 2014	Jeroen Ooms (2014)	https://arxiv.org/abs/1403.2805
Linear graph	dplyr	Working with data frame like objects.	v 1.0.5	Hadley Wickham *et al.* (2021)	https://cran.r-project.org/web/packages/dplyr
	ggpubr	'ggplot2' based publication ready plots.	v 0.4.0	Alboukadel Kassambara (2020)	https://cran.r-project.org/web/packages/ggpubr
Boxplot	dplyr	Working with data frame like objects.	v 1.0.5	Hadley Wickham *et al.* (2021)	https://cran.r-project.org/web/packages/dplyr
	ggpubr	'ggplot2' based publication ready plots.	v 0.4.0	Alboukadel Kassambara (2020)	https://cran.r-project.org/web/packages/ggpubr
Corrplot	corrplot	Visualization of a correlation matrix.	v 0.84	Taiyun Wei and Viliam Simko (2017)	https://cran.r-project.org/web/packages/corrplot
	dplyr	Working with data frame like objects.	v 1.0.5	Hadley Wickham *et al.* (2021)	https://cran.r-project.org/web/packages/dplyr
	ggplot2	Elegant graphics for data analysis.	v 2016	H. Wickham (2016)	https://ggplot2.tidyverse.org
GO enrichment	clusterProfiler	Statistical analysis and visualization of functional profiles for genes and gene clusters.	v 3.18.1	Guangchuang Yu *et al.* (2012); PMID: 22455463	http://www.bioconductor.org/packages/release/bioc/html/clusterProfiler.html
	dplyr	Working with data frame like objects.	v 1.0.5	Hadley Wickham *et al.*	https://cran.r-project.org/web/p

		objects.		(2021)	ackages/dplyr
	ggplot2	Elegant graphics for data analysis.	v 2016	H. Wickham (2016)	https://ggplot2.tidyverse.org
	jsonlite	A practical and consistent mapping between JSON data and R objects.	v 2014	Jeroen Ooms (2014)	https://arxiv.org/abs/1403.2805
	org.Hs.eg.db	Genome wide annotation for Human.	v 3.11.4	Marc Carlson (2020)	http://www.bioconductor.org/packages/release/data/annotation/html/org.Hs.eg.db.html
	Rgraphviz	Graph for R objects.	v 2.32.0	Kasper Daniel Hansen *et al.* (2020)	http://www.bioconductor.org/packages/release/bioc/html/Rgraphviz.html
	stringr	Common string operations.	v 1.4.0	Hadley Wickham (2019)	https://cran.r-project.org/web/packages/stringr
	topGO	Enrichment analysis for Gene Ontology.	v 2.40.0	Adrian Alexa and Jorg Rahnenfuhrer (2020)	http://www.bioconductor.org/packages/release/bioc/html/topGO.html
KEGG enrichment	clusterProfiler	Statistical analysis and visualization of functional profiles for genes and gene clusters.	v 3.18.1	Guangchuang Yu *et al.* (2012); PMID: 22455463	http://www.bioconductor.org/packages/release/bioc/html/clusterProfiler.html
	dplyr	Working with data frame like objects.	v 1.0.5	Hadley Wickham *et al.* (2021)	https://cran.r-project.org/web/packages/dplyr
	ggplot2	Elegant graphics for data analysis.	v 2016	H. Wickham (2016)	https://ggplot2.tidyverse.org
	jsonlite	A practical and consistent mapping between JSON data and R objects.	v 2014	Jeroen Ooms (2014)	https://arxiv.org/abs/1403.2805
	org.Hs.eg.db	Genome wide annotation for Human.	v 3.11.4	Marc Carlson (2020)	http://www.bioconductor.org/packages/release/data/annotation/html/org.Hs.eg.db.html
	pathview	Pathway-based data integration and visualization.	v 1.30.1	Luo Weijun and Brouwer Cory (2014); PMID: 23740750	http://www.bioconductor.org/packages/release/bioc/html/pathview.html
	Rgraphviz	Graph for R objects.	v 2.32.0	Kasper Daniel Hansen *et al.* (2020)	http://www.bioconductor.org/packages/release/bioc/html/Rgraphviz.html
	stringr	Common string operations.	v 1.4.0	Hadley Wickham (2019)	https://cran.r-project.org/web/packages/stringr
**Tools**					
Web Service	clusterProfiler	Statistical analysis and visualization of functional profiles for genes and gene clusters.	v 3.18.1	Guangchuang Yu *et al.* (2012); PMID: 22455463	http://www.bioconductor.org/packages/release/bioc/html/clusterProfiler.html
	dplyr	Working with data frame like objects.	v 1.0.5	Hadley Wickham *et al.* (2021)	https://cran.r-project.org/web/packages/dplyr
	GenomicFeatures	Software for computing and annotating genomic ranges.	v 2013	Michael Lawrence *et al.* (2013); PMID:	http://bioconductor.org/packages/release/bioc/html/Genomic

				23950696	Features.html
	jsonlite	A practical and consistent mapping between JSON data and R objects.	1.44.0	Jeroen Ooms (2014)	https://arxiv.org/abs/1403.2805
	org.Hs.eg.db	Genome wide annotation for Human.	v 3.11.4	Marc Carlson (2020)	http://www.bioconductor.org/packages/release/data/annotation/html/org.Hs.eg.db.html
	tidyverse	R packages for data science.	v 1.31	Hadley Wickham *et al.* 2019	https://cran.r-project.org/web/packages/tidyverse/
Drug Discovery	clusterProfiler	Statistical analysis and visualization of functional profiles for genes and gene clusters.	v 3.18.1	Guangchuang Yu *et al.* (2012); PMID: 22455463	http://www.bioconductor.org/packages/release/bioc/html/clusterProfiler.html
	data.table	Extension of "data.frame".	v 1.14.0	Matt Dowle *et al*. 2021	https://cran.r-project.org/web/packages/data.table/
	dplyr	Working with data frame like objects.	v 1.0.5	Hadley Wickham *et al.* (2021)	https://cran.r-project.org/web/packages/dplyr
	jsonlite	A practical and consistent mapping between JSON data and R objects.	v 2014	Jeroen Ooms (2014)	https://arxiv.org/abs/1403.2805
	org.Hs.eg.db	Genome wide annotation for Human.	v 3.11.4	Marc Carlson (2020)	http://www.bioconductor.org/packages/release/data/annotation/html/org.Hs.eg.db.html
	pathview	Pathway-based data integration and visualization.	v 1.30.1	Luo Weijun and Brouwer Cory (2014); PMID: 23740750	http://www.bioconductor.org/packages/release/bioc/html/pathview.html
Gene Conversion	clusterProfiler	Statistical analysis and visualization of functional profiles for genes and gene cluster.	v 3.18.1	Guangchuang Yu *et al.* (2012); PMID: 22455463	http://www.bioconductor.org/packages/release/bioc/html/clusterProfiler.html
	jsonlite	A practical and consistent mapping between JSON data and R objects.	v 2014	Jeroen Ooms (2014)	https://arxiv.org/abs/1403.2805
	org.Hs.eg.db	Genome wide annotation for Human.	v 3.11.4	Marc Carlson (2020)	http://www.bioconductor.org/packages/release/data/annotation/html/org.Hs.eg.db.html
	tidyverse	R packages for data science.	v 1.31	Hadley Wickham *et al.* 2019	https://cran.r-project.org/web/packages/tidyverse/index.html

Supplementary Table S3. The details of the resources used in COVID19db

Resource	Description	Version	URL
DrugCentral	A database to provide information on active ingredients chemical entities, pharmaceutical products, drug mode of action, indications, pharmacologic action.	v. 17 May 2020	https://drugcentral.org/
GENCODE	A resource for for integrated annotation of gene features.	v. December 2020	https://www.gencodegenes.org/
KEGG PATHWAY Database	A database to provide a collection of manually drawn pathway maps representing our knowledge of the molecular interaction, reaction and relation networks.	v. 3 June 2021	https://www.kegg.jp/kegg/pathway.html
NCBI GEO	A archive for functional genomics data sets-update.	v. 1 July 2021	https://www.ncbi.nlm.nih.gov/geo/
R	A free software environment for statistical computing and graphics.	v. 4.0.3	https://www.r-project.org/

Supplementary Table S4. The details of the four potential drugs and their targets

Drug name	Target name	Target class	Target pathway name: KEGG ID	Action value	Action type	Action comment
bortezomib	ELANE	Enzyme	Systemic lupus erythematosus: hsa05322	5.64	Ki	Inhibitory activity against human leukocyte elastase
boceprevir	ELANE	Enzyme	Systemic lupus erythematosus: hsa05322	4.77	IC50	Inhibition of human leukocyte elastase after 60 mins fluorescence assay
sivelestat	ELANE	Enzyme	Systemic lupus erythematosus: hsa05322	6.7	Ki	Binding affinity against Elastase
telaprevir	ELANE	Enzyme	Systemic lupus erythematosus: hsa05322	6.17	IC50	Inhibition of human leukocyte elastase after 60 mins fluorescence assay

Supplementary Table S5. The comparison of the data contents and utilities of COVID19db with the CovidExpress database

**Data contents and utilities**	**COVID19db**	**CovidExpress**
**Data contents**		
The number of transcriptomes	4127	1093
The number of datasets	95	35
The number of microbes	33	7
The number of drugs/agents	33	11
Drug-target-pathway interactions	39,930 interactions among 2037 drugs, 1116 targets, and 207 pathways	None
Annotations for samples and datasets	Yes (more annotations, including age, gender, treatment conditions, and virus strain)	Yes
**Utilities**		
**(1) Differential expression analysis**		
Gene expression overview	Yes (heatmap, PCA, and box plot)	Yes (PCA, Histogram, DotPlot, and track plot)
Overall differential gene expression analysis	Yes	Yes
Differential expression analysis on single gene	Yes (box plot)	Yes (violin plot)
Volcano plot	Yes	Yes
Heatmap plot on the custom genes	Yes	Yes
GO enrichment analysis on the differential genes	Yes	None
KEGG enrichment analysis on the differential genes	Yes	None
Pathway view on the differential genes	Yes	None
GSEA analysis on the differential genes	None	Yes
Clustering analysis	PCA and heatmap	Yes (PCA, tSNE, and UMAP)
**(2) Co-expression analysis**		
Co-expression analysis on an interesting gene with all genes	Yes	None
Co-expression analysis on two genes (unpaired)	Yes	Yes
Co-expression analysis on two genes (paired)	Yes	None
Co-expression analysis on multiple genes	Yes	None
GO enrichment analysis on the	Yes	None

co-expression genes		
KEGG enrichment analysis on the co-expression genes	Yes	None
**(3) Tool**		
Drug discovery tool	Yes (The Drug Discovery tool)	None
Analysis on researchers' own data	Yes (The Web Service tool)	None
New data submission for further integration	Yes	None
Gene name conversion	Yes (The Gene Conversion tool)	None
Data download freely	Yes	Yes
**(4) Support**		
Environment and other tools support	No special need	Yes (Python 3.6+ and the cellxgene tool)

Note: PCA - Principal Component Analysis; GO - Gene Ontology; GSEA - Gene Set Enrichment Analysis; KEGG - Kyoto Encyclopedia of Genes and Genomes; tSNE - t-distributed Stochastic Neighbor Embedding; UMAP - Uniform Manifold Approximation and Projection.
